# Geographical Variation in Psychiatric Admissions Among Recipients of Public Assistance

**DOI:** 10.2188/jea.JE20180066

**Published:** 2019-07-05

**Authors:** Yasuyuki Okumura, Nobuo Sakata, Hisateru Tachimori, Tadashi Takeshima

**Affiliations:** 1 Research Department, Institute for Health Economics and Policy, Association for Health Economics Research and Social Insurance and Welfare, Tokyo, Japan; 2 Department of Psychiatry and Behavioral Science, Tokyo Metropolitan Institute of Medical Science, Tokyo, Japan; 3 National Institute of Mental Health, National Center of Neurology and Psychiatry, Tokyo, Japan; 4 Kawasaki City Center for Mental Health and Welfare, Kanagawa, Japan

**Keywords:** spatial variation, ecological study, supplier-induced demand, drift effects, breeder effects

## Abstract

**Background:**

Understanding the area-specific resource use of inpatient psychiatric care is essential for the efficient use of the public assistance system. This study aimed to assess the geographical variation in psychiatric admissions and to identify the prefecture-level determinants of psychiatric admissions among recipients of public assistance in Japan.

**Methods:**

We identified all recipients of public assistance who were hospitalized in a psychiatric ward in May 2014, 2015, or 2016 using the Fact-finding Survey on Medical Assistance. The age- and sex-standardized number of psychiatric admissions was calculated for each of the 47 prefectures, using direct and indirect standardization methods.

**Results:**

A total of 46,559 psychiatric inpatients were identified in May 2016. The number of psychiatric admissions per 100,000 population was 36.6. We found a 7.1-fold difference between the prefectures with the highest (Nagasaki) and lowest (Nagano) numbers of admissions. The method of decomposing explained variance in the multiple regression model showed that the number of psychiatric beds per 100,000 population and the number of recipients of public assistance per 1,000 population were the most important determinants of the number of psychiatric admissions (*R*^2^ = 28% and *R*^2^ = 23%, respectively). The sensitivity analyses, using medical cost as the outcome and data from different survey years and subgroups, showed similar findings.

**Conclusions:**

We identified a large geographical variation in the number and total medical cost of psychiatric admissions among recipients of public assistance. Our findings should encourage policy makers to assess the rationale for this variation and consider strategies for reducing it.

## INTRODUCTION

Japan’s public assistance system is aimed at guaranteeing a minimum level of healthy and cultural life and at enhancing independence for all citizens with hardship.^1^^,^^2^ The system consists of eight types of assistance programs, including those for livelihood, housing, education, medical, long-term care, maternity, occupational, and funeral assistance.^1^^,^^2^ Almost all recipients of public assistance can receive medical care without out-of-pocket expenditure.

In 2015, there were approximately 2 million recipients of public assistance in Japan,^3^ a country with a population of 127 million.^4^ The annual cost of medical assistance was about 1.8 trillion yen (17 billion United States dollars) in 2015. The inpatient cost for mental disorders accounted for 15% of the total medical cost in recipients of medical assistance, whereas the proportion was much smaller for the entire population (4%).^5^^,^^6^ The national government suggests that approximately 20% of patients hospitalized at psychiatric wards should be discharged from the hospitals when the conditions (eg, living accommodation/consultation services) for community-based care are given.^6^ To reduce potentially avoidable long-term stays in psychiatric wards, local governments have implemented various programs for promoting the discharge from the hospital to the community among recipients with mental disorders.^6^ Understanding the area-specific resource use for inpatient psychiatric care is essential for the reduction of potentially avoidable long-term stays and can thus contribute to the appropriate use of the public assistance system through enhancement of independence rather than institutionalization. Local governments will assess the rationale for the number of psychiatric admissions in their own prefectures in comparison to that of Japan as a whole. Moreover, the national government will need to judge whether a geographical variation in the number of psychiatric admissions appears to be justified based on the aims of the public assistance system.

To date, there is no information on the area-specific number of psychiatric admissions among recipients of public assistance. We aimed at assessing the geographical variation in the number of psychiatric admissions and at identifying the prefecture-level determinants of psychiatric admissions among recipients of public assistance in Japan.

## METHODS

### Study design and setting

We conducted an ecological study using data from the Fact-finding Survey on Medical Assistance (FSMA). A detailed description of the FSMA has been reported elsewhere.^5^ The FSMA is conducted every year by the Ministry of Health, Labour and Welfare in Japan. It collects all electronically issued medical claims reviewed every June (ie, claims for medical care in April and May) among all recipients of medical assistance. Since 2014, data of the FSMA include parameters, such as prefectural identification numbers based on the location of the residence, patient identification numbers, diagnostic codes, procedural codes, length of the hospital stay, and total medical cost per month. We obtained anonymous patient-level data from the FSMA conducted in 2014, 2015, and 2016.

The Japanese local government system is composed of 47 prefectural governments. There is a large heterogeneity in the prefecture-level potential determinants of resource use for inpatient psychiatric care (eTable 1). For example, the number of recipients of public assistance per 1,000 population was 16.7 in 2015 (ie, 2,127,841 recipients in all of Japan); this varied by prefecture, ranging from 3.2 in Toyama to 33.3 in Osaka in 2015.^3^

### Patient selection

All claims of patients hospitalized at a psychiatric ward for at least 1 day during May of the study year (2014, 2015, and 2016) were identified. To avoid duplicates, we included only claims for psychiatric care provided in May. For patients who were hospitalized at more than two hospitals in May, we included one claim, issued from the hospital with the longer stay.

### Statistical analyses

In the main analysis, we used data from the FSMA, 2016. First, we calculated the age- and sex-standardized number and total medical cost of psychiatric admissions for each of the 47 prefectures, using direct and indirect standardization methods.^7^ Aggregated data from the Population Census in 2015 were used as the standard population.^4^ We selected the unit of population as 100,000 population for the direct standardization method. In the analyses of the number of psychiatric admissions, we used the standardized claim ratio (SCR), which is analogous to the standardized mortality ratio and enables the comparison of the number of psychiatric admissions in a specific prefecture to that in Japan as a whole (reference standard).^8^^,^^9^ The empirical Bayes estimates for the SCRs were obtained using the Poisson-Gamma model along with the moment estimator^10^; the 95% equal-tail credible intervals (CrIs) for the SCRs were obtained using posterior distribution.^11^ In the analyses of the total medical cost, we used the multiplicative effect (ME), which also enables the comparison of the total medical costs in a specific prefecture to that in Japan as a whole (reference standard). We obtained the estimates for the MEs using the Poisson model along with the natural logarithm of the number of the population as an offset term; the 95% confidence intervals (CIs) for the MEs were obtained using the robust standard error.^12^

Second, we investigated the associations between the age- and sex-standardized direct estimates and a set of prefecture-level potential determinants using multiple regression models. We selected the following ecological factors as potential determinants based on a priori clinical knowledge, previous studies, and availability of data (eTable 1)^13^^–^^15^: (1) the number of recipients of public assistance per 1,000 population in 2015,^3^^,^^4^ (2) the number of treated patients with mental disorders per 1,000 population in 2014,^16^ (3) the number of visits for psychiatric nursing care at home per 100,000 population in 2014,^4^^,^^17^ (4) the number of psychiatric beds per 100,000 population in 2016,^18^ (5) the average length of stay in psychiatric beds in 2016,^18^ (6) the number of beds in institutions covered by long-term care insurance per 1,000 population aged ≥65 years in 2015,^4^^,^^19^ (7) the percentage of depopulated municipalities in 2014,^20^ (8) the percentage of population living alone in 2015,^21^ and (9) unemployment rate.^20^^,^^22^ Multicollinearity was assessed by examining Pearson correlation coefficients between all pairs of the independent variables, tolerance, and variance inflation factor (VIF) values. As a rule of thumb, tolerance values <0.1 or VIF statistics >10 indicates that potentially harmful multicollinearity is present.^23^ We simultaneously entered all independent variables into the models and obtained the Lindeman-Merenda-Gold metrics to quantify the amount of explained variance (overall *R*^2^) attributable to each independent variable (*R*^2^) in the multiple regression model.^24^ The sum of *R*^2^ for all independent variables is equal to the overall *R*^2^ in the model.

The significance level was set at 5% for all analyses. All data were analyzed using R version 3.3.2 (R Foundation for Statistical Computing, Vienna, Austria).

### Additional analyses

We also conducted three sets of additional analyses. First, we repeated the main analyses using data from the 2014 and 2015 FSMA to investigate the robustness of the study findings.

Second, we conducted the following subgroup analyses for the number of psychiatric admissions to assess the effect of the type of psychiatric admission: (1) patients experiencing a new long-stay (length of stay between ≥1 year and <5 years), (2) those experiencing an old long-stay (length of stay ≥5 years), and (3) those having a primary diagnosis of dementia (ICD-10 codes F00–F03 and G30–G31). Old long-stay patients were defined as those who had been admitted to a psychiatric bed before deinstitutionalization and remained hospitalized, while new long-stay patients were defined as those who began prolonged hospitalization despite efforts to prevent it.^25^

Third, we compared the number of psychiatric admissions by prefecture between the FSMA (2016) and the 630 survey (2015) to examine the generalizability of the study results.^26^^,^^27^ The 630 survey collects the number of “all” psychiatric admissions by prefecture, sex, age group, diagnostic group, and length of stay on June 30; however, this survey does not collect information on the status of public assistance. In this analysis, we calculated the age- and sex-standardized number of psychiatric admissions per 100,000 population using the direct method. The outcomes included the standardized number of (1) psychiatric admissions, (2) psychiatric admissions for a new long-stay, (3) psychiatric admissions for an old long-stay, and (4) dementia-related psychiatric admissions. Then, we computed Pearson correlation coefficients.

### Ethics

The Statistics Act regulates primary data collection and the secondary use of data. Because all patient-level data were certified to be anonymous, our study design was not subject to the ethical guideline for medical research in Japan.^28^ Institutional review board approval was not required according to the guideline of the local ethics committee.

## RESULTS

### Study population

**Figure 1.**
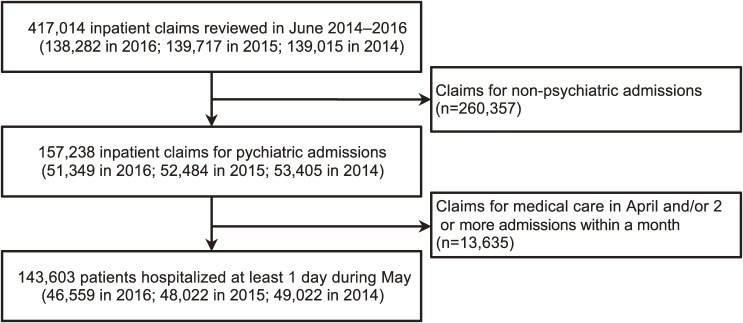
Flow diagram of the study population

A total of 46,559 psychiatric inpatients (33.7% of the 138,282 potentially eligible admissions) were included in the main analyses (Figure 1). Of the entire sample, 20,701 (44.5%) were women. The median age was 64 years (interquartile range, 53–72 years) for the entire sample, 63 years (interquartile range, 52–71 years) for new long-stay patients, 67 years (interquartile range, 60–74 years) for old long-stay patients, and 78 years (interquartile range, 72–84 years) for dementia patients. A total of 31,834 inpatients (68.4%) were hospitalized for at least 1 year, and 4,422 (9.5%) had a primary diagnosis of dementia (eTable 2). The median total medical cost per month was 372,250 yen (interquartile range, 339,640–398,900 yen).

### Geographical variation in the resource use for psychiatric admissions and determinants

**Figure 2.**
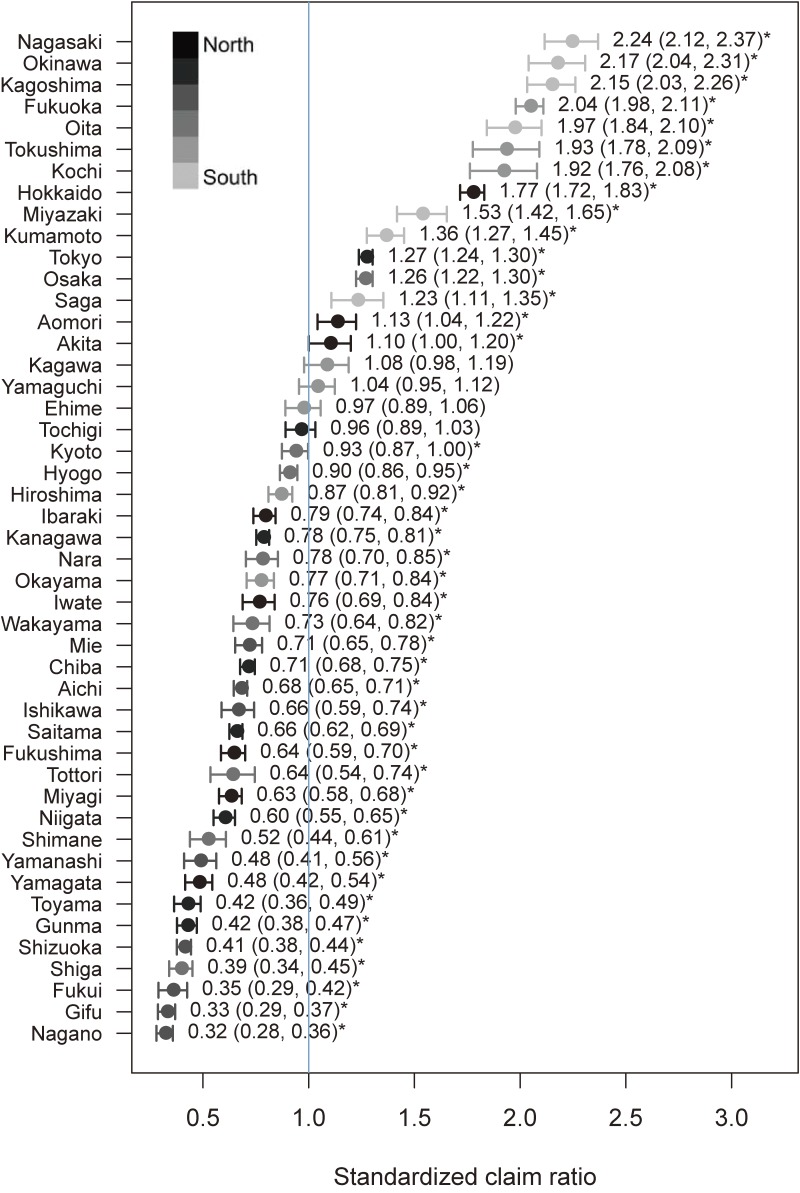
Age- and sex-standardized claim ratio of the number of psychiatric admissions among recipients of public assistance for each of the 47 prefectures. ^*^Indicates a statistically significant difference from Japan’s national mean

The number of psychiatric admissions per 100,000 population in 2016 was 36.6. There was a 7.1-fold difference between the prefectures with the highest (Nagasaki) and lowest (Nagano) numbers of admissions (direct method: 83.3 and 12.0 admissions per 100,000 population in Nagasaki and Nagano, respectively; indirect method: 2.24 in Nagasaki and 0.32 in Nagano; Figure 2 and eTable 3). The numbers of psychiatric admissions in the seven prefectures located in the south of Japan (ie, Nagasaki, Okinawa, Kagoshima, Fukuoka, Oita, Tokushima, and Kochi) were approximately two times higher than that in Japan as a whole (Figure 2 and eTable 3).

Our findings on the geographical variation were consistent across outcomes. The total medical cost of psychiatric admissions per month per 100,000 population was 13.8 million yen. We found a 7.4-fold difference between the prefectures with the highest (Okinawa) and lowest (Fukui) costs (direct method: 3,098,708 and 425,078 yen per 100,000 population in Okinawa and in Fukui, respectively; indirect method: 2.70 in Okinawa and 0.36 in Fukui; eTable 4).

**Figure 3.**
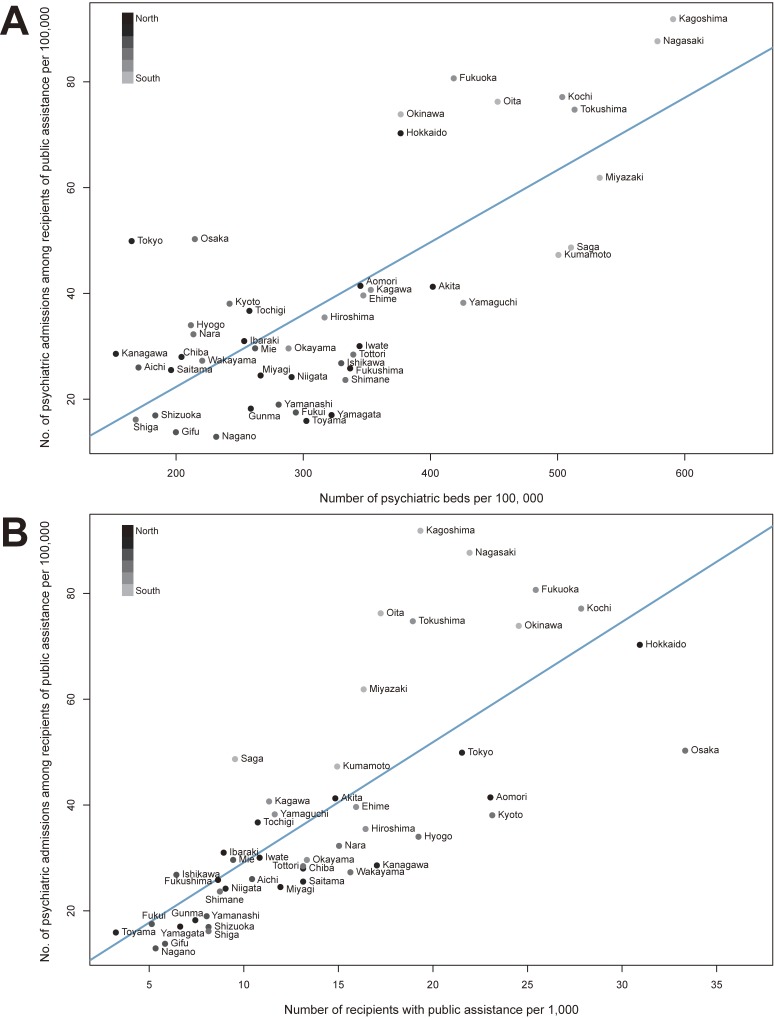
Scatterplot of the age- and sex-standardized number of psychiatric admissions among recipients of public assistance per 100,000 population vs the number of psychiatric beds per 100,000 population (A); and vs the number of recipients of public assistance per 1,000 population (B).

Correlation coefficients between all pairs of the independent variables were ≤0.75, the tolerance values were ≥0.17, and the VIF statistics were ≤5.75, suggesting that multicollinearity is not a substantive problem (eTable 5). The multiple regression model revealed that the number of psychiatric beds per 100,000 population and the number of recipients of public assistance per 1,000 population were the most important determinants of the number of psychiatric admissions (*R*^2^ = 28% and *R*^2^ = 23%, respectively; eTable 6). For each 100-unit increase in the number of psychiatric beds per 100,000 population, the number of psychiatric admission increased by 11 admissions per 100,000 population (Figure 3A and eTable 6). For each 10-unit increase in the number of recipients of public assistance per 1,000 population, the number of psychiatric admission increased by 13 admissions per 100,000 population (Figure 3B and eTable 6).

Similar findings were observed for the total medical cost of psychiatric admissions (eTable 7). For each 100-unit increase in the number of psychiatric beds per 100,000 population, the total medical cost of psychiatric admissions increased by 4,178,500 yen per month per 100,000 population (eTable 7). For each 10-unit increase in the number of recipients of public assistance per 1,000 population, the total medical cost of psychiatric admission increased by 5,078,680 yen per month per 100,000 population (eTable 7).

### Analysis by survey year

We then repeated the above analyses using data from the FSMA, 2014 and 2015. In the sensitivity analyses, the total number of included patients was 49,022 in 2014 and 48,022 in 2015 (Figure 1 and eTable 2). The results of the sensitivity analyses were similar to those of the main analyses (eTable 3, eTable 4, eTable 6, and eTable 7).

### Subgroup analysis of the number of psychiatric admissions for a new long-stay

We observed an 8.0-fold difference between the prefectures with the highest (Kochi) and lowest (Gifu) numbers of admissions for a new long-stay (direct method: 20.6 and 2.4 admissions per 100,000 population in Kochi and Gifu, respectively; indirect method: 2.18 in Kochi and 0.27 in Gifu; eTable 8). The numbers of psychiatric admissions in the five prefectures located in the south of Japan (ie, Kochi, Fukuoka, Okinawa, Nagasaki, and Kagoshima) were approximately two times higher than that in Japan as a whole (eTable 8).

The multiple regression model showed that the number of psychiatric beds per 100,000 population and the number of recipients of public assistance per 1,000 population were the most important determinants of psychiatric admissions for a new long-stay (*R*^2^ = 27% and *R*^2^ = 24%, respectively; eTable 9).

### Subgroup analysis of the number of psychiatric admissions for an old long-stay

We also observed a large geographical variation for the number of psychiatric admissions for an old long-stay (eTable 10). We found an 11.7-fold difference between the prefectures with the highest (Nagasaki) and lowest (Nagano) numbers of admissions (direct method: 44.5 and 3.7 admissions per 100,000 population in Nagasaki and in Nagano, respectively; indirect method: 2.77 in Nagasaki and 0.24 in Nagano; eTable 10).

Unlike the findings from the main analyses, the multiple regression model showed that the number of psychiatric beds per 100,000 population was the most important determinant of the number of psychiatric admissions for an old long-stay (*R*^2^ = 30%); this was followed by the number of recipients of public assistance per 1,000 population (*R*^2^ = 17%) and the average length of stay in psychiatric beds (*R*^2^ = 17%; eTable 11).

### Subgroup analysis of the number of dementia-related psychiatric admissions

The number of dementia-related psychiatric admissions showed marked geographical variation (eTable 12). There was a 29.2-fold difference between the prefectures with the highest (Okinawa) and lowest (Nagano) numbers of dementia-related psychiatric admissions (direct method: 9.8 and 0.3 per 100,000 in Okinawa and in Nagano, respectively; indirect method: 2.73 in Okinawa and 0.09 in Nagano).

Unlike the findings of the main analyses, the multiple regression model showed that the number of recipients of public assistance per 1,000 population was the most important determinant of the number of dementia-related psychiatric admissions (*R*^2^ = 25%); this was followed by unemployment rate (*R*^2^ = 20%) and the number of psychiatric beds per 100,000 population (*R*^2^ = 15%; eTable 13).

### Differences in the numbers of psychiatric admissions between the FSMA and the 630 survey

**Figure 4.**
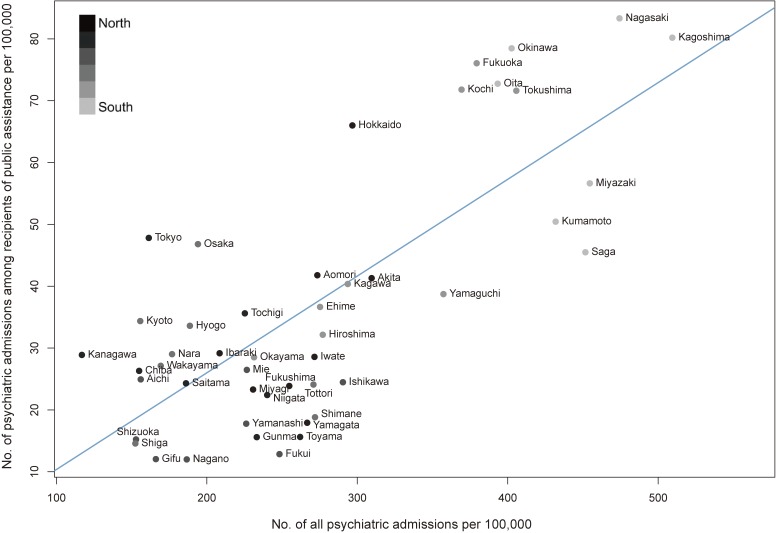
Scatterplot of the age- and sex-standardized number of psychiatric admissions among recipients of public assistance per 100,000 population vs the age- and sex-standardized number of all psychiatric admissions per 100,000 population

We observed strong positive correlations for all outcomes between the FSMA and the 630 survey (Figure 4 and eTable 14): the number of psychiatric admissions (*r* = 0.75), number of psychiatric admissions for a new long-stay (*r* = 0.74), number of psychiatric admissions for an old long-stay (*r* = 0.80), and number of dementia-related psychiatric admissions (*r* = 0.68).

## DISCUSSION

We found an over 7-fold geographic variation in the number and total medical cost of psychiatric admissions among recipients of public assistance between prefectures. Similar to our results, some previous studies in smaller areas found significant geographical variations in the numbers of psychiatric admission.^29^^–^^32^ For example, Watts et al found a 6-fold variation in the psychiatric admission rate by hospital service area in Northern New England.^29^ Fortney et al also found a substantial geographic variation in the county-level hospitalization rate for schizophrenia in 14 states of the United States.^30^ Our study is unique as it focused on recipients of public assistance in all of Japan.

We observed that psychiatric admissions occurred more frequently in prefectures where the number of recipients of public assistance per population is higher. The simple reason for this association is that such prefectures are likely to have a greater demand for psychiatric inpatient care. Of note, in Japan, recipients of public assistance commonly suffer from mental illnesses (14% of all recipients).^3^ Namely, the number of recipients of public assistance might be a proxy measure for the number of recipients with mental illnesses. Consequently, prefectures with a higher number of recipients of public assistance per population may have a greater demand for psychiatric admissions among recipients of public assistance. Another reason for this association is that case workers in prefectures with a higher caseload are less likely to be motivated to promote the patients’ discharge from hospitals to the community than those in prefectures with a lower caseload. This is because, in general, caseloads are lower for recipients receiving long-term inpatient care than those receiving ambulatory care.

We found a significant association between the number of psychiatric admissions among recipients of public assistance and the number of psychiatric beds per population on the prefectural level, after controlling for several ecological factors. A similar association was observed for the number of psychiatric admissions not only for an old but also for a new long-stay; however, this association was weaker for dementia-related psychiatric admissions. The reason for the association among old long-stay patients is interpreted as a reflection of the “historical” phenomenon. Historically, the number of psychiatric beds in Japan steeply increased from 95,067 in 1960 to 247,265 in 1970, and then steadily increased to 333,570 by 1985.^33^ During the era of growing number of psychiatric beds, public assistance was a common expenditure programs for psychiatric inpatient care. The percentage of psychiatric admissions covered by public assistance relative to all psychiatric admissions ranged from 32% to 50% between 1960 and 1985.^33^^,^^34^ It can be assumed that most old long-stay patients in our study were hospitalized from this era, which was partially supported by the fact that over half of old-stay patients were aged ≥65 years.

On the contrary, the reason for the association among new long-stay patients may be interpreted as a reflection of the “supplier-induced demand” phenomenon,^29^^,^^30^ which is considered likely to occur in psychiatric admissions.^29^^,^^30^^,^^35^ Demand of inpatient psychiatric care might be induced until beds are full, irrespective of necessity. This is because physicians are in a strong position in which they can define the patients’ needs for the initiation and termination of inpatient psychiatric care. Given strong concerns by citizens and policy makers regarding the potential overuse of medical assistance, our findings should be of interest to local governments, particularly to those with high psychiatric admission rates for a new long-stay.

We identified a strong positive correlation in psychiatric admission rates between the FSMA and the 630 survey, suggesting that our results of the prefecture-level determinants of psychiatric admissions may be generalizable to all psychiatric inpatients. Namely, the extent and mechanism of the geographical variations in psychiatric admissions may not be unique to recipients of public assistance.

Our findings have several limitations. First, our results were inherently vulnerable to confounding and ecological biases due to the nature of the study design. Second, we could not determine whether the admission was involuntary or not, because all claims data from admission to discharge are needed to identify the admission status. Involuntary admissions may be more associated with psychiatric bed availability than voluntary admissions. This is because the most common type of involuntary admission called the “hospitalization for medical care and protection” strongly considers the opinions of the patients’ family regarding admission and discharge, and they often prefer hospitalization for longer period. Third, we could not identify the exact length of hospitalization due to the nature of the data structure. Fourth, we could not incorporate ambulatory psychiatric services into our analyses because the medical costs for ambulatory services in recipients of public assistance who have chronic psychiatric illnesses are likely supported by another healthcare system (the “Medical System for Services and Supports for Persons with Disabilities”) rather than medical assistance.

### Conclusion

We found a large geographical variation in the number and total medical cost of psychiatric admissions among recipients of public assistance. The number of recipients of public assistance per population and psychiatric bed availability may explain this variation. Our findings should encourage policy makers to assess the rationale for and consider strategies for reducing this variation.

## APPENDIX A. SUPPLEMENTARY DATA

eTable 1. Prefecture-level characteristics

eTable 2. Characteristics of study population

eTable 3. Age- and sex-standardized number of psychiatric admissions by prefecture and survey year, using direct and indirect methods

eTable 4. Age- and sex-standardized total medical costs of psychiatric admissions by prefecture and survey year using direct and indirect methods

eTable 5. Correlations matrix and variance inflation factor

eTable 6. Potential determinants of the number of psychiatric admissions per 100,000 population by survey year

eTable 7. Potential determinants of the total cost of psychiatric admissions per 100,000 population by survey year

eTable 8. Age- and sex-standardized number of new long-stay psychiatric patients by prefecture and survey year, using direct and indirect methods

eTable 9. Potential determinants of the number of new long-stay psychiatric patients per 100,000 population by survey year

eTable 10. Age- and sex-standardized number of old long-stay psychiatric patients by prefecture and survey year, using direct and indirect methods

eTable 11. Potential determinants of the number of old long-stay psychiatric patients per 100,000 population

eTable 12. Age- and sex-standardized number of dementia-related psychiatric admissions by prefecture and survey year, using direct and indirect methods

eTable 13. Potential determinants of the number of dementia-related psychiatric admissions per 100,000 population

eTable 14. Differences in psychiatric admission data between the FSMA and the 630 survey
