# Supplementary material for: Geographical Variation in Psychiatric Admissions Among Recipients of Public Assistance
Source: J Epidemiol. 2019 Jul 5;29(7):264–71. doi: 10.2188/jea.JE20180066 (PMC6556440; doi:10.2188/jea.JE20180066)
Supplement: Supplementary file 1 [file je-29-264-s001.pdf]

eTable 1. Prefecture-level characteristics

Prefecture	Population, n	Recipients of public assistance per 1,000 population, n	Treated patients with mental disorders per 1,000 population, n	Visits for psychiatric nursing care at home per 100,000 population, n	Psychiatric beds per 100,000 population, n	Average length of stay in psychiatric beds, days
Japan	127,094,745	16.7	30.9	102.3	263.3	269.9
Hokkaido	5,381,733	30.9	31.8	283.6	376.0	261.0
Aomori	1,308,265	23.0	36.7	166.3	344.4	233.8
Iwate	1,279,594	10.8	25.0	106.4	343.7	262.5
Miyagi	2,333,899	11.9	39.0	74.3	265.9	288.7
Akita	1,023,119	14.8	31.3	51.7	401.3	269.2
Yamagata	1,123,891	6.6	37.4	89.7	321.7	234.1
Fukushima	1,914,039	8.6	35.0	88.9	336.3	314.7
Ibaraki	2,916,976	8.9	35.0	46.4	253.0	321.8
Tochigi	1,974,255	10.7	21.3	108.4	257.1	348.9
Gunma	1,973,115	7.4	27.9	69.6	258.2	322.5
Saitama	7,266,534	13.1	22.3	48.2	195.5	271.6
Chiba	6,222,666	13.1	21.1	49.0	203.7	312.3
Tokyo	13,515,271	21.5	40.3	57.6	164.5	193.1
Kanagawa	9,126,214	17.0	27.9	63.4	152.1	229.3
Niigata	2,304,264	9.0	25.2	74.1	290.3	325.3
Toyama	1,066,328	3.2	20.6	152.5	301.9	292.9
Ishikawa	1,154,008	6.4	24.3	113.4	329.3	274.2
Fukui	786,740	5.1	20.3	88.7	293.6	243.9
Yamanashi	834,930	8.0	34.7	109.2	280.1	254.2
Nagano	2,098,804	5.3	41.5	53.4	231.0	222.1
Gifu	2,031,903	5.8	33.5	74.9	199.3	256.6
Shizuoka	3,700,305	8.1	28.9	57.9	183.1	266.9
Aichi	7,483,128	10.4	29.8	76.2	169.9	250.0
Mie	1,815,865	9.4	25.9	123.3	261.6	310.5
Shiga	1,412,916	8.1	26.2	35.7	167.7	251.3
Kyoto	2,610,353	23.1	29.5	88.8	241.5	255.0
Osaka	8,839,469	33.3	31.7	81.4	214.2	228.9
Hyogo	5,534,800	19.2	24.2	43.9	211.1	264.2
Nara	1,364,316	15.0	24.9	55.9	213.1	251.4
Wakayama	963,579	15.6	29.1	221.1	220.0	306.8
Tottori	573,441	13.1	40.1	209.8	338.8	273.4
Shimane	694,352	8.7	41.8	132.1	332.6	244.6
Okayama	1,921,525	13.3	42.2	62.4	287.9	227.2
Hiroshima	2,843,990	16.4	30.2	210.0	316.2	289.9
Yamaguchi	1,404,729	11.6	27.1	288.4	425.3	395.3
Tokushima	755,733	18.9	49.0	162.6	512.8	355.2
Kagawa	976,263	11.3	42.0	110.4	352.6	312.1
Ehime	1,385,262	15.9	51.3	192.2	346.7	313.4
Kochi	728,276	27.8	41.2	81.8	503.1	231.2
Fukuoka	5,101,556	25.4	27.2	139.9	417.8	296.4
Saga	832,832	9.5	30.0	254.7	510.0	298.0
Nagasaki	1,377,187	21.9	37.8	146.2	578.0	354.3
Kumamoto	1,786,170	14.9	32.5	165.8	500.1	268.9
Oita	1,166,338	17.2	35.2	173.3	452.3	415.2
Miyazaki	1,104,069	16.3	28.1	366.8	532.6	335.2
Kagoshima	1,648,177	19.3	32.8	270.3	590.3	361.1
Okinawa	1,433,566	24.5	24.4	225.0	376.1	253.5

(eTable 1 continues)

(eTable 1 continued)

Prefecture	Beds in institutions covered by long-term care insurance per 1,000 population aged ≥65 years, n	Depopulated municipalities, %	Population living alone, %	Unemployment rate, %
Japan	15.5	46.4	14.5	3.1
Hokkaido	15.0	83.2	16.9	3.6
Aomori	14.1	70.0	11.7	4.0
Iwate	17.9	66.7	11.6	2.4
Miyagi	15.0	25.7	13.9	3.2
Akita	19.5	84.0	10.6	3.2
Yamagata	21.8	60.0	8.9	2.4
Fukushima	18.2	49.2	11.7	2.6
Ibaraki	18.0	9.1	10.9	2.9
Tochigi	14.1	12.0	11.1	2.7
Gunma	17.2	40.0	11.2	2.5
Saitama	15.9	6.3	12.4	3.1
Chiba	13.5	11.1	13.5	2.9
Tokyo	14.1	15.4	23.4	3.2
Kanagawa	15.2	0.0	15.4	3.1
Niigata	20.6	46.7	10.1	2.8
Toyama	16.6	20.0	9.6	2.3
Ishikawa	19.4	47.4	12.4	2.3
Fukui	19.3	35.3	9.4	1.9
Yamanashi	15.1	55.6	11.7	2.6
Nagano	17.7	48.1	10.7	2.5
Gifu	17.0	33.3	9.5	2.4
Shizuoka	16.3	22.9	11.0	2.5
Aichi	12.0	9.3	13.7	2.4
Mie	17.0	31.0	11.6	2.0
Shiga	15.2	10.5	10.8	2.5
Kyoto	15.4	34.6	16.9	3.1
Osaka	13.1	2.3	16.6	4.0
Hyogo	14.8	22.0	13.7	3.4
Nara	17.0	38.5	10.0	3.2
Wakayama	18.0	60.0	11.9	2.0
Tottori	17.9	63.2	11.1	2.4
Shimane	21.4	100.0	11.5	1.7
Okayama	17.6	74.1	12.9	2.7
Hiroshima	14.1	69.6	14.7	2.7
Yamaguchi	14.1	63.2	14.2	2.4
Tokushima	15.2	54.2	13.0	2.7
Kagawa	16.9	47.1	12.9	2.6
Ehime	14.7	85.0	14.3	2.6
Kochi	17.2	82.4	15.9	3.3
Fukuoka	15.3	35.0	16.1	3.5
Saga	14.9	45.0	9.7	2.1
Nagasaki	15.3	61.9	12.9	2.8
Kumamoto	14.4	60.0	12.2	3.2
Oita	13.7	88.9	13.8	2.6
Miyazaki	16.9	65.4	13.4	2.3
Kagoshima	19.7	95.3	15.6	2.8
Okinawa	15.8	43.9	12.6	4.4

eTable 2. Characteristics of study population

Characteristic	Survey year 2016 (n=46,559) n	%	2015 (n=48,022) n	%	2014 (n=49,022) n	%
Type of hospital						
General hospital	1,061	2.3	1,122	2.3	1,051	2.1
Other	45,498	97.7	46,900	97.7	47,971	97.9
Type of hospital fee						
Fee-for-service plan (patient-to-nurse-ratio)						
Psychiatric unit (10:1)	85	0.2	84	0.2	114	0.2
Psychiatric unit (13:1)	518	1.1	571	1.2	394	0.8
Psychiatric unit (15:1)	22,012	47.3	22,498	46.8	23,085	47.1
Psychiatric unit (18:1)	1,029	2.2	1,210	2.5	1,534	3.1
Psychiatric unit (20:1)	296	0.6	340	0.7	466	1.0
Specialized psychiatric unit	308	0.7	393	0.8	360	0.7
Fee-for-service plan in advanced treatment hospitals (patient-to-nurse-ratio)						
Psychiatric unit (7:1)	14	0.0	17	0.0	11	0.0
Psychiatric unit (10:1)	53	0.1	39	0.1	39	0.1
Psychiatric unit (13:1)	83	0.2	78	0.2	78	0.2
Psychiatric unit (15:1)	23	0.0	36	0.1	31	0.1
Per-diem payment plan						
Psychiatric emergency unit	1,213	2.6	1,127	2.3	970	2.0
Psychiatric acute care unit	2,186	4.7	2,239	4.7	2,091	4.3
Psychiatric emergency and physical complication unit	34	0.1	40	0.1	18	0.0
Child and adolescent psychiatric unit	58	0.1	43	0.1	38	0.1
Chronic psychiatric care unit	15,850	34.0	16,442	34.2	16,887	34.4
Dementia care unit	2,797	6.0	2,865	6.0	2,906	5.9
Sex						
Men	25,858	55.5	26,752	55.7	27,484	56.1
Women	20,701	44.5	21,270	44.3	21,538	43.9
Age, years						
0–19	168	0.4	142	0.3	153	0.3
20–39	3,083	6.6	3,306	6.9	3,465	7.1
40–64	21,111	45.3	22,128	46.1	22,917	46.7
65–74	13,457	28.9	13,755	28.6	13,668	27.9
≥75	8,740	18.8	8,691	18.1	8,819	18.0
Primary diagnosis for mental disorders (ICD-10 codes)[a]						
Alcohol use disorder (F10)	3,571	7.7	3,703	7.7	3,807	7.8
Drug use disorders (F11–F16, F18–F19)	511	1.1	517	1.1	488	1.0
Schizophrenia (F2)	28,956	62.2	29,916	62.3	30,719	62.7
Unipolar depressive disorders (F32–F33, F341)	1,771	3.8	1,835	3.8	1,829	3.7
Bipolar affective disorders (F30–F31)	1,555	3.3	1,639	3.4	1,571	3.2
Anxiety disorders (F40–F44)	675	1.5	670	1.4	650	1.3
Eating disorders (F50)	43	0.1	51	0.1	43	0.1
Pervasive and developmental disorders (F840, F845)	43	0.1	46	0.1	25	0.1
Childhood behavioral disorders (F7, F90–F92)	472	1.0	415	0.9	365	0.7
Other mental disorders	2,225	4.8	2,157	4.5	2,289	4.7
Primary diagnosis of dementia (ICD-10 codes: F00–F03, G30–G31)	4,422	9.5	4,507	9.4	4,578	9.3
Length of hospital stay						
<3 months	7,385	15.9	7,602	15.8	7,423	15.1
≥3 months to <1 year	7,340	15.8	7,274	15.1	7,382	15.1
≥1 year to <5 years	11,737	25.2	12,188	25.4	12,379	25.3
≥5 years	20,097	43.2	20,958	43.6	21,838	44.5
Total medical cost per month, median yen (interquartile range)	372,250 (339,640–398,900)		369,680 (337,540–395,300)		369,680 (336,622–396,200)	

[a] Diagnostic codes were based on the Global Burden of Disease Study 2010.

eTable 3. Age- and sex-standardized number of psychiatric admissions by prefecture and survey year, using direct and indirect methods

	Direct method, number of admissions per 100,000 population			Indirect method, standardized claim ratio (95% CrI)		
Prefecture	2016	2015	2014	2016	2015	2014
Japan	36.6	37.8	38.6	reference	reference	reference
Hokkaido	66.0	67.6	70.2	1.77 (1.72, 1.83)*	1.76 (1.71, 1.82)*	1.79 (1.74, 1.85)*
Aomori	41.7	45.3	41.4	1.13 (1.04, 1.22)*	1.17 (1.09, 1.27)*	1.06 (0.97, 1.14)
Iwate	28.5	31.4	30.0	0.76 (0.69, 0.84)*	0.80 (0.72, 0.88)*	0.76 (0.68, 0.83)*
Miyagi	23.3	24.6	24.4	0.63 (0.58, 0.68)*	0.64 (0.59, 0.70)*	0.62 (0.57, 0.68)*
Akita	41.3	39.3	41.2	1.10 (1.00, 1.20)*	1.02 (0.93, 1.12)	1.05 (0.96, 1.15)
Yamagata	17.9	17.8	17.0	0.48 (0.42, 0.54)*	0.47 (0.41, 0.53)*	0.44 (0.38, 0.50)*
Fukushima	23.8	23.4	25.8	0.64 (0.59, 0.70)*	0.61 (0.56, 0.67)*	0.65 (0.60, 0.71)*
Ibaraki	29.1	29.1	30.9	0.79 (0.74, 0.84)*	0.76 (0.71, 0.82)*	0.80 (0.75, 0.85)*
Tochigi	35.6	36.7	36.7	0.96 (0.89, 1.03)	0.96 (0.89, 1.03)	0.94 (0.88, 1.01)
Gunma	15.6	15.8	18.2	0.42 (0.38, 0.47)*	0.41 (0.37, 0.46)*	0.47 (0.42, 0.52)*
Saitama	24.3	25.2	25.5	0.66 (0.62, 0.69)*	0.67 (0.63, 0.70)*	0.66 (0.63, 0.69)*
Chiba	26.3	28.5	28.0	0.71 (0.68, 0.75)*	0.75 (0.71, 0.78)*	0.72 (0.68, 0.75)*
Tokyo	47.8	49.9	49.9	1.27 (1.24, 1.30)*	1.28 (1.25, 1.31)*	1.26 (1.22, 1.29)*
Kanagawa	28.9	29.0	28.5	0.78 (0.75, 0.81)*	0.76 (0.73, 0.79)*	0.73 (0.70, 0.76)*
Niigata	22.4	23.1	24.1	0.60 (0.55, 0.65)*	0.60 (0.55, 0.65)*	0.61 (0.56, 0.66)*
Toyama	15.6	14.5	15.9	0.42 (0.36, 0.49)*	0.38 (0.32, 0.44)*	0.41 (0.35, 0.47)*
Ishikawa	24.4	24.4	26.8	0.66 (0.59, 0.74)*	0.64 (0.57, 0.72)*	0.69 (0.62, 0.77)*
Fukui	12.8	13.9	17.5	0.35 (0.29, 0.42)*	0.37 (0.30, 0.44)*	0.46 (0.39, 0.54)*
Yamanashi	17.7	21.5	18.9	0.48 (0.41, 0.56)*	0.56 (0.49, 0.65)*	0.49 (0.42, 0.57)*
Nagano	12.0	12.0	12.9	0.32 (0.28, 0.36)*	0.31 (0.27, 0.35)*	0.32 (0.29, 0.36)*
Gifu	12.0	12.7	13.7	0.33 (0.29, 0.37)*	0.33 (0.30, 0.38)*	0.35 (0.31, 0.40)*
Shizuoka	15.2	15.6	16.9	0.41 (0.38, 0.44)*	0.41 (0.37, 0.44)*	0.43 (0.40, 0.47)*
Aichi	24.9	25.6	26.0	0.68 (0.65, 0.71)*	0.68 (0.65, 0.71)*	0.67 (0.64, 0.70)*
Mie	26.4	27.8	29.6	0.71 (0.65, 0.78)*	0.73 (0.67, 0.79)*	0.76 (0.70, 0.82)*
Shiga	14.5	16.1	16.1	0.39 (0.34, 0.45)*	0.42 (0.37, 0.48)*	0.41 (0.36, 0.47)*
Kyoto	34.3	37.4	38.0	0.93 (0.87, 1.00)*	0.98 (0.92, 1.05)	0.98 (0.92, 1.04)
Osaka	46.8	48.8	50.2	1.26 (1.22, 1.30)*	1.28 (1.24, 1.31)*	1.29 (1.25, 1.33)*
Hyogo	33.6	35.1	33.9	0.90 (0.86, 0.95)*	0.92 (0.88, 0.96)*	0.87 (0.83, 0.91)*
Nara	29.0	29.2	32.2	0.78 (0.70, 0.85)*	0.76 (0.69, 0.84)*	0.82 (0.75, 0.90)*
Wakayama	27.1	26.6	27.2	0.73 (0.64, 0.82)*	0.70 (0.62, 0.78)*	0.70 (0.62, 0.78)*
Tottori	24.1	24.5	28.4	0.64 (0.54, 0.74)*	0.64 (0.54, 0.75)*	0.72 (0.61, 0.83)*
Shimane	18.8	20.5	23.6	0.52 (0.44, 0.61)*	0.54 (0.45, 0.63)*	0.60 (0.51, 0.69)*
Okayama	28.5	30.0	29.6	0.77 (0.71, 0.84)*	0.79 (0.72, 0.85)*	0.76 (0.70, 0.82)*
Hiroshima	32.1	32.5	35.4	0.87 (0.81, 0.92)*	0.85 (0.79, 0.90)*	0.91 (0.85, 0.96)*
Yamaguchi	38.7	37.9	38.2	1.04 (0.95, 1.12)	0.99 (0.91, 1.07)	0.98 (0.90, 1.06)
Tokushima	71.6	71.4	74.7	1.93 (1.78, 2.09)*	1.86 (1.71, 2.02)*	1.91 (1.76, 2.07)*
Kagawa	40.3	39.6	40.6	1.08 (0.98, 1.19)	1.03 (0.93, 1.13)	1.03 (0.94, 1.14)
Ehime	36.6	39.3	39.6	0.97 (0.89, 1.06)	1.01 (0.93, 1.09)	1.00 (0.92, 1.09)
Kochi	71.8	76.5	77.1	1.92 (1.76, 2.08)*	1.99 (1.84, 2.16)*	1.96 (1.81, 2.12)*
Fukuoka	76.0	78.3	80.6	2.04 (1.98, 2.11)*	2.04 (1.98, 2.11)*	2.06 (1.99, 2.12)*
Saga	45.5	46.5	48.6	1.23 (1.11, 1.35)*	1.21 (1.09, 1.33)*	1.24 (1.12, 1.37)*
Nagasaki	83.3	84.6	87.6	2.24 (2.12, 2.37)*	2.21 (2.09, 2.34)*	2.24 (2.11, 2.36)*
Kumamoto	50.4	46.9	47.2	1.36 (1.27, 1.45)*	1.24 (1.15, 1.32)*	1.21 (1.13, 1.30)*
Oita	72.7	74.1	76.2	1.97 (1.84, 2.10)*	1.95 (1.82, 2.08)*	1.95 (1.83, 2.08)*
Miyazaki	56.6	62.4	61.8	1.53 (1.42, 1.65)*	1.63 (1.51, 1.75)*	1.58 (1.47, 1.70)*
Kagoshima	80.2	85.3	91.8	2.15 (2.03, 2.26)*	2.24 (2.12, 2.35)*	2.34 (2.22, 2.46)*
Okinawa	78.4	72.0	73.8	2.17 (2.04, 2.31)*	1.94 (1.82, 2.07)*	1.94 (1.82, 2.07)*

CrI, credible interval.

*Indicates a statistically significant difference from Japan's national mean

eTable 4. Age- and sex-standardized total medical costs of psychiatric admissions by prefecture and survey year using direct and indirect methods

Prefecture	Direct method, costs per month per 100,000 population (Japanese yen)			Indirect method, multiplicative effect (95% CI)		
	2016	2015	2014	2016	2015	2014
Japan	13,781,489	14,078,804	14,226,207	reference	reference	reference
Hokkaido	25,026,870	25,418,300	26,184,110	2.12 (2.02, 2.23)*	2.12 (2.02, 2.23)*	2.13 (2.02, 2.25)*
Aomori	14,940,300	16,352,640	14,357,170	1.28 (1.19, 1.37)*	1.36 (1.25, 1.48)*	1.17 (1.09, 1.26)*
Iwate	10,561,730	11,063,640	10,530,570	0.88 (0.77, 1.01)	0.90 (0.78, 1.03)	0.85 (0.77, 0.94)*
Miyagi	8,780,533	9,116,270	9,011,531	0.74 (0.69, 0.81)*	0.76 (0.69, 0.84)*	0.73 (0.65, 0.82)*
Akita	14,642,890	13,715,980	14,174,880	1.24 (1.13, 1.36)*	1.14 (1.03, 1.26)*	1.16 (1.07, 1.26)*
Yamagata	7,595,429	6,788,425	6,242,322	0.64 (0.53, 0.77)*	0.56 (0.47, 0.68)*	0.51 (0.44, 0.60)*
Fukushima	8,733,251	8,479,678	9,198,535	0.74 (0.67, 0.81)*	0.70 (0.63, 0.78)*	0.74 (0.67, 0.82)*
Ibaraki	9,885,284	9,733,355	10,312,850	0.84 (0.78, 0.91)*	0.82 (0.75, 0.89)*	0.84 (0.78, 0.91)*
Tochigi	12,835,370	13,017,090	13,013,320	1.09 (1.02, 1.16)*	1.09 (1.00, 1.19)*	1.06 (0.98, 1.16)
Gunma	5,777,945	5,841,379	6,491,284	0.49 (0.43, 0.56)*	0.49 (0.44, 0.54)*	0.53 (0.48, 0.59)*
Saitama	9,191,140	9,509,933	9,588,116	0.78 (0.73, 0.83)*	0.80 (0.74, 0.87)*	0.78 (0.72, 0.85)*
Chiba	9,806,037	10,587,760	10,198,460	0.83 (0.78, 0.89)*	0.88 (0.84, 0.93)*	0.83 (0.79, 0.87)*
Tokyo	18,333,600	19,024,080	18,858,240	1.53 (1.43, 1.64)*	1.56 (1.45, 1.68)*	1.51 (1.41, 1.62)*
Kanagawa	10,855,230	10,675,710	10,470,610	0.93 (0.87, 0.98)*	0.89 (0.85, 0.94)*	0.85 (0.81, 0.90)*
Niigata	7,950,355	8,316,223	8,560,402	0.67 (0.61, 0.73)*	0.69 (0.63, 0.74)*	0.69 (0.63, 0.76)*
Toyama	5,729,391	5,178,156	5,645,694	0.49 (0.41, 0.58)*	0.43 (0.36, 0.51)*	0.46 (0.38, 0.54)*
Ishikawa	9,450,332	9,366,522	10,064,310	0.80 (0.70, 0.93)*	0.78 (0.66, 0.93)*	0.82 (0.72, 0.94)*
Fukui	4,250,780	4,827,967	5,934,504	0.36 (0.30, 0.44)*	0.40 (0.33, 0.48)*	0.49 (0.41, 0.59)*
Yamanashi	6,643,804	7,882,143	6,504,432	0.56 (0.49, 0.65)*	0.66 (0.59, 0.73)*	0.53 (0.46, 0.62)*
Nagano	4,598,932	4,260,866	4,594,430	0.38 (0.32, 0.46)*	0.35 (0.29, 0.41)*	0.36 (0.30, 0.44)*
Gifu	4,294,193	4,626,641	4,791,732	0.36 (0.33, 0.41)*	0.39 (0.34, 0.44)*	0.39 (0.34, 0.45)*
Shizuoka	5,719,913	5,843,555	6,224,670	0.48 (0.44, 0.53)*	0.49 (0.45, 0.53)*	0.51 (0.47, 0.55)*
Aichi	9,259,240	9,600,954	9,555,090	0.79 (0.71, 0.88)*	0.81 (0.74, 0.88)*	0.79 (0.70, 0.89)*
Mie	9,842,139	10,430,300	11,290,540	0.83 (0.77, 0.91)*	0.87 (0.80, 0.95)*	0.92 (0.86, 0.98)*
Shiga	5,425,304	5,954,347	6,066,896	0.46 (0.40, 0.53)*	0.49 (0.43, 0.57)*	0.49 (0.41, 0.58)*
Kyoto	12,716,310	13,581,530	13,422,710	1.09 (0.97, 1.22)	1.14 (1.02, 1.26)*	1.10 (0.99, 1.23)
Osaka	18,206,500	18,621,470	18,810,710	1.55 (1.47, 1.63)*	1.55 (1.47, 1.64)*	1.54 (1.46, 1.61)*
Hyogo	12,876,300	13,399,500	12,741,540	1.09 (1.04, 1.15)*	1.12 (1.05, 1.19)*	1.04 (0.97, 1.11)
Nara	11,321,660	11,030,790	12,265,050	0.95 (0.84, 1.08)	0.92 (0.82, 1.03)	0.99 (0.88, 1.12)
Wakayama	9,244,243	9,536,979	9,601,252	0.78 (0.70, 0.87)*	0.79 (0.73, 0.87)*	0.78 (0.72, 0.85)*
Tottori	9,505,344	9,112,663	10,146,560	0.78 (0.68, 0.91)*	0.76 (0.65, 0.89)*	0.82 (0.72, 0.94)*
Shimane	7,141,159	7,612,556	8,682,998	0.61 (0.50, 0.75)*	0.63 (0.57, 0.71)*	0.70 (0.61, 0.81)*
Okayama	10,834,120	11,621,320	11,529,500	0.92 (0.86, 0.99)*	0.97 (0.89, 1.07)	0.94 (0.86, 1.04)
Hiroshima	12,084,230	12,273,230	13,155,720	1.02 (0.96, 1.09)	1.02 (0.94, 1.10)	1.07 (0.99, 1.16)
Yamaguchi	14,536,480	13,733,930	13,875,750	1.23 (1.10, 1.37)*	1.14 (1.02, 1.28)*	1.13 (0.98, 1.30)
Tokushima	26,489,400	25,566,740	27,041,960	2.27 (2.05, 2.51)*	2.14 (1.94, 2.37)*	2.22 (2.04, 2.42)*
Kagawa	15,374,730	14,282,600	14,359,430	1.30 (1.18, 1.43)*	1.19 (1.08, 1.30)*	1.17 (1.07, 1.27)*
Ehime	13,512,280	14,117,880	14,385,580	1.13 (1.01, 1.26)*	1.16 (1.04, 1.28)*	1.16 (1.06, 1.26)*
Kochi	27,558,900	28,877,330	28,463,940	2.33 (2.16, 2.51)*	2.42 (2.22, 2.64)*	2.32 (2.10, 2.55)*
Fukuoka	29,058,610	29,877,200	30,523,310	2.46 (2.34, 2.59)*	2.49 (2.36, 2.63)*	2.48 (2.36, 2.61)*
Saga	17,212,680	17,074,090	17,991,570	1.46 (1.33, 1.61)*	1.42 (1.27, 1.58)*	1.46 (1.29, 1.66)*
Nagasaki	30,332,570	30,272,430	31,828,840	2.58 (2.39, 2.78)*	2.54 (2.31, 2.80)*	2.60 (2.41, 2.80)*
Kumamoto	17,164,300	16,547,570	17,239,500	1.46 (1.35, 1.59)*	1.40 (1.30, 1.50)*	1.41 (1.29, 1.54)*
Oita	26,983,440	27,651,520	28,010,720	2.31 (2.12, 2.52)*	2.33 (2.12, 2.56)*	2.29 (2.12, 2.47)*
Miyazaki	21,021,750	23,301,660	22,440,030	1.80 (1.66, 1.96)*	1.94 (1.78, 2.12)*	1.83 (1.70, 1.98)*
Kagoshima	28,567,940	29,979,610	32,054,940	2.42 (2.26, 2.59)*	2.52 (2.35, 2.71)*	2.61 (2.46, 2.78)*
Okinawa	30,987,080	27,729,810	27,763,180	2.70 (2.46, 2.96)*	2.40 (2.13, 2.70)*	2.34 (2.10, 2.60)*

*Indicates a statistically significant difference from Japan's national mean

CI, confidence interval

eTable 5. Correlations matrix and variance inflation factor

Independent variable	Pearson correlation coefficient										
	1	2	3	4	5	6	7	8	9	Tolerance	VIF
1. Number of recipients of public assistance per 1,000 population	—									0.17	5.75
2. Number of treated patients with mental disorders per 1,000 population	0.17	—								0.61	1.63
3. Number of patients receiving home visits for psychiatric nursing care per 100,000 population	0.27	0.06	—							0.39	2.54
4. Number of psychiatric beds per 100, 000 population	0.28	0.28	0.66	—						0.31	3.27
5. Average length of stay in psychiatric beds, days	−0.09	−0.05	0.45	0.50	—					0.56	1.78
6. Number of beds in institutions covered by long-term care insurance per 1,000 population aged ≥65 years	−0.41	0.05	−0.08	0.12	−0.08	—				0.51	1.98
7. Percentage of depopulated municipalities	0.16	0.47	0.53	0.68	0.22	0.36	—			0.31	3.24
8. Percentage of population living alone	0.71	0.22	0.12	0.03	−0.10	−0.46	−0.02	—		0.38	2.60
9. Unemployment rate	0.75	−0.01	−0.08	0.01	−0.26	−0.40	−0.14	0.44	—	0.29	3.44

VIF, variance inflation factor.

eTable 6. Potential determinants of the number of psychiatric admissions per 100,000 population by survey year

	2016 (Overall R^2=0.91)			2015 (Overall R^2=0.92)			2014 (Overall R^2=0.92)		
Independent variable	r	B (95% CI)	$R^{2\,a}$	r	B (95% CI)	$R^{2\,a}$	r	B (95% CI)	$R^{2\,a}$
Intercept	—	–55.30 (–97.43, –13.16)*	—	—	–49.90 (–90.02, –9.78)*	—	—	–57.84 (–100.24, –15.44)*	—
1. Number of recipients of public assistance per 1,000 population	0.76	1.33 (0.64, 2.03)*	0.23	0.77	1.49 (0.83, 2.15)*	0.24	0.75	1.61 (0.91, 2.30)*	0.24
2. Number of treated patients with mental disorders per 1,000 population	0.21	–0.02 (–0.37, 0.33)	0.01	0.22	–0.05 (–0.38, 0.29)	0.01	0.22	–0.04 (–0.40, 0.31)	0.01
3. Number of patients receiving home visits for psychiatric nursing care per 100,000 population	0.50	–0.02 (–0.06, 0.03)	0.07	0.50	–0.02 (–0.06, 0.02)	0.07	0.51	–0.02 (–0.06, 0.02)	0.07
4. Number of psychiatric beds per 100, 000 population	0.73	0.11 (0.08, 0.15)*	0.28	0.73	0.12 (0.09, 0.15)*	0.27	0.74	0.12 (0.09, 0.15)*	0.28
5. Average length of stay in psychiatric beds, days	0.34	0.07 (0.01, 0.12)*	0.06	0.34	0.06 (0.01, 0.12)*	0.06	0.36	0.08 (0.02, 0.13)*	0.07
6. Number of beds in institutions covered by long-term care insurance per 1,000 population aged ≥65 years	–0.26	0.05 (–1.24, 1.35)	0.02	–0.26	–0.01 (–1.25, 1.22)	0.03	–0.23	0.39 (–0.91, 1.70)	0.02
7. Percentage of depopulated municipalities	0.37	–0.10 (–0.24, 0.04)	0.05	0.38	–0.09 (–0.22, 0.04)	0.05	0.39	–0.11 (–0.24, 0.03)	0.05
8. Percentage of population living alone	0.50	1.07 (–0.19, 2.32)	0.09	0.52	1.27 (0.07, 2.46)*	0.10	0.51	1.30 (0.04, 2.56)*	0.10
9. Unemployment rate	0.49	3.60 (–3.20, 10.39)	0.10	0.47	1.12 (–5.35, 7.59)	0.09	0.44	0.08 (–6.76, 6.92)	0.08

CI, confidence interval.

* P<0.05

a R^2 indicates a decomposition of overall R^2 in the multiple regression model. The sum of R^2 for all independent variables is equal to the overall R^2 in the model.

eTable 7. Potential determinants of the total cost of psychiatric admissions per 100,000 population by survey year

	2016 (Overall R^2=0.90)			2015 (Overall R^2=0.91)			2014 (Overall R^2=0.91)		
Independent variable	r	B (95% CI)	$R^{2\,a}$	r	B (95% CI)	$R^{2\,a}$	r	B (95% CI)	$R^{2\,a}$
Intercept	—	−20,680,553 (−37,437,806, −3,923,300)*	—	—	−17,041,780 (−32,608,027, −1,475,532)*	—	—	−19,068,202 (−35,352,617, −2,783,787)*	—
1. Number of recipients of public assistance per 1,000 population	0.77	507,868 (231,489, 784,247)*	0.24	0.78	561,664 (304,928, 818,400)*	0.25	0.76	603,151 (334,570, 871,732)*	0.24
2. Number of treated patients with mental disorders per 1,000 population	0.21	−1,981 (−141,487, 137,525)	0.01	0.21	−29,710 (−159,301, 99,881)	0.01	0.21	−24,658 (−160,228, 110,912)	0.01
3. Number of patients receiving home visits for psychiatric nursing care per 100,000 population	0.49	−3,631 (−20,165, 12,904)	0.07	0.50	−6,858 (−22,217, 8,501)	0.07	0.50	−78,97 (−23,964, 8,171)	0.07
4. Number of psychiatric beds per 100, 000 population	0.71	41,785 (29,114, 54,456)*	0.26	0.71	41,521 (29,751, 53,291)*	0.26	0.73	43,624 (31,310, 55,937)*	0.27
5. Average length of stay in psychiatric beds, days	0.31	21,348 (−1,547, 44,243)	0.05	0.31	20,014 (−1,254, 41,282)	0.05	0.34	24,010 (1,761, 46,260)*	0.06
6. Number of beds in institutions covered by long-term care insurance per 1,000 population aged ≥65 years	−0.27	52,177 (−463,164, 567,517)	0.03	−0.28	−19,272 (−497,985, 459,441)	0.03	−0.25	93,451 (−407,348, 594,249)	0.02
7. Percentage of depopulated municipalities	0.36	−41,026 (−95,582, 13,530)	0.04	0.37	−30,735 (−81,413, 19,943)	0.05	0.38	−39,740 (−92,757, 13,276)	0.05
8. Percentage of population living alone	0.51	403,291 (−94,870, 901,452)	0.09	0.54	491,283 (28,529, 954,038)*	0.11	0.52	489,211 (5,106, 973,315)*	0.10
9. Unemployment rate	0.50	1,480,578 (−1,221,334, 4,182,490)	0.11	0.49	435,652 (−2,074,224, 2,945,528)	0.10	0.45	−115,046 (−2,740,718, 2,510,626)	0.08

CI, confidence interval.
* *P*<0.05
a R^2 indicates a decomposition of overall R^2 in the multiple regression model. The sum of R^2 for all independent variables is equal to the overall R^2 in the model.

eTable 8. Age- and sex-standardized number of new long-stay psychiatric patients by prefecture and survey year, using direct and indirect methods

Prefecture	Direct method, no. of admissions per 100,000 population			Indirect method, standardized claim ratio (95% CrI)		
	2016	2015	2014	2016	2015	2014
Japan	9.2	9.6	9.7	reference	reference	reference
Hokkaido	17.1	16.8	16.5	1.83 (1.71, 1.94)*	1.73 (1.63, 1.85)*	1.68 (1.57, 1.79)*
Aomori	11.0	11.8	10.7	1.17 (1.00, 1.36)	1.21 (1.03, 1.40)*	1.07 (0.90, 1.24)
Iwate	7.7	7.9	8.0	0.83 (0.68, 0.99)*	0.80 (0.66, 0.96)*	0.80 (0.66, 0.95)*
Miyagi	7.6	7.9	8.4	0.81 (0.69, 0.93)*	0.81 (0.70, 0.94)*	0.85 (0.74, 0.98)*
Akita	9.7	9.0	8.9	0.99 (0.82, 1.19)	0.91 (0.75, 1.10)	0.90 (0.73, 1.08)
Yamagata	5.2	4.9	4.1	0.57 (0.44, 0.71)*	0.52 (0.40, 0.66)*	0.43 (0.32, 0.55)*
Fukushima	6.4	6.7	7.3	0.69 (0.58, 0.81)*	0.69 (0.58, 0.81)*	0.74 (0.62, 0.86)*
Ibaraki	6.9	6.9	7.2	0.74 (0.64, 0.84)*	0.71 (0.62, 0.81)*	0.73 (0.64, 0.83)*
Tochigi	7.7	9.6	9.7	0.82 (0.70, 0.96)*	0.99 (0.85, 1.13)	0.98 (0.85, 1.13)
Gunma	3.5	3.5	4.3	0.39 (0.30, 0.48)*	0.37 (0.29, 0.46)*	0.44 (0.35, 0.54)*
Saitama	5.7	5.9	6.0	0.61 (0.56, 0.67)*	0.61 (0.56, 0.67)*	0.61 (0.56, 0.67)*
Chiba	6.2	6.3	6.3	0.67 (0.60, 0.74)*	0.65 (0.59, 0.72)*	0.65 (0.59, 0.71)*
Tokyo	13.0	13.7	13.7	1.36 (1.29, 1.43)*	1.37 (1.30, 1.44)*	1.35 (1.29, 1.42)*
Kanagawa	8.4	8.3	8.0	0.90 (0.83, 0.96)*	0.85 (0.79, 0.91)*	0.81 (0.76, 0.88)*
Niigata	6.0	6.0	6.4	0.64 (0.54, 0.74)*	0.61 (0.51, 0.71)*	0.64 (0.54, 0.74)*
Toyama	3.4	3.5	3.5	0.38 (0.27, 0.50)*	0.37 (0.27, 0.49)*	0.38 (0.27, 0.50)*
Ishikawa	6.4	5.6	5.3	0.68 (0.54, 0.85)*	0.59 (0.46, 0.74)*	0.56 (0.43, 0.70)*
Fukui	3.5	3.1	3.7	0.40 (0.27, 0.56)*	0.34 (0.22, 0.48)*	0.41 (0.28, 0.56)*
Yamanashi	2.9	3.6	2.8	0.33 (0.22, 0.46)*	0.39 (0.27, 0.53)*	0.31 (0.21, 0.44)*
Nagano	3.3	3.6	4.3	0.36 (0.28, 0.44)*	0.36 (0.29, 0.45)*	0.42 (0.34, 0.51)*
Gifu	2.4	2.6	2.9	0.27 (0.21, 0.35)*	0.27 (0.21, 0.35)*	0.30 (0.23, 0.38)*
Shizuoka	3.9	4.0	4.5	0.42 (0.36, 0.49)*	0.42 (0.36, 0.49)*	0.46 (0.39, 0.53)*
Aichi	6.1	6.8	6.9	0.66 (0.60, 0.72)*	0.71 (0.65, 0.78)*	0.71 (0.65, 0.78)*
Mie	5.6	6.8	6.8	0.60 (0.49, 0.72)*	0.70 (0.59, 0.83)*	0.69 (0.58, 0.82)*
Shiga	2.8	3.9	4.2	0.32 (0.23, 0.42)*	0.42 (0.32, 0.54)*	0.44 (0.34, 0.56)*
Kyoto	7.6	7.4	8.7	0.83 (0.72, 0.95)*	0.78 (0.67, 0.89)*	0.90 (0.78, 1.02)
Osaka	11.3	12.8	12.7	1.21 (1.13, 1.28)*	1.32 (1.25, 1.40)*	1.29 (1.21, 1.37)*
Hyogo	8.3	8.6	8.3	0.89 (0.81, 0.97)*	0.88 (0.81, 0.96)*	0.84 (0.77, 0.92)*
Nara	6.8	7.1	7.5	0.72 (0.58, 0.87)*	0.73 (0.59, 0.88)*	0.76 (0.62, 0.91)*
Wakayama	6.2	5.5	5.9	0.67 (0.51, 0.84)*	0.56 (0.43, 0.72)*	0.60 (0.46, 0.76)*
Tottori	6.5	8.8	7.9	0.70 (0.50, 0.93)*	0.91 (0.69, 1.16)	0.80 (0.59, 1.03)
Shimane	6.1	6.0	6.7	0.67 (0.49, 0.87)*	0.65 (0.48, 0.84)*	0.70 (0.53, 0.90)*
Okayama	7.5	7.8	7.5	0.80 (0.68, 0.94)*	0.80 (0.68, 0.93)*	0.76 (0.64, 0.89)*
Hiroshima	7.9	8.4	10.0	0.85 (0.74, 0.96)*	0.87 (0.76, 0.98)*	1.01 (0.89, 1.13)
Yamaguchi	11.3	10.8	11.4	1.20 (1.02, 1.38)*	1.11 (0.95, 1.29)	1.16 (0.99, 1.33)
Tokushima	16.3	15.5	15.8	1.68 (1.41, 1.98)*	1.54 (1.28, 1.82)*	1.55 (1.29, 1.83)*
Kagawa	8.6	7.7	7.8	0.92 (0.74, 1.12)	0.81 (0.65, 1.00)	0.80 (0.64, 0.99)*
Ehime	10.0	10.8	10.9	1.04 (0.88, 1.22)	1.07 (0.91, 1.25)	1.10 (0.93, 1.27)
Kochi	20.6	21.4	20.8	2.18 (1.86, 2.52)*	2.15 (1.84, 2.49)*	2.07 (1.77, 2.39)*
Fukuoka	19.8	20.9	21.4	2.11 (1.98, 2.24)*	2.14 (2.01, 2.27)*	2.15 (2.02, 2.28)*
Saga	12.0	12.6	13.9	1.28 (1.04, 1.54)*	1.29 (1.06, 1.54)*	1.37 (1.14, 1.63)*
Nagasaki	19.2	19.9	18.8	2.04 (1.81, 2.28)*	2.03 (1.80, 2.27)*	1.86 (1.65, 2.09)*
Kumamoto	11.6	11.2	10.6	1.23 (1.07, 1.40)*	1.16 (1.00, 1.32)*	1.08 (0.94, 1.24)
Oita	15.9	14.9	15.6	1.69 (1.46, 1.93)*	1.54 (1.32, 1.77)*	1.58 (1.36, 1.80)*
Miyazaki	14.7	13.5	14.0	1.56 (1.33, 1.80)*	1.40 (1.19, 1.62)*	1.43 (1.22, 1.66)*
Kagoshima	18.3	20.4	22.4	1.94 (1.73, 2.16)*	2.08 (1.87, 2.30)*	2.25 (2.03, 2.48)*
Okinawa	19.3	18.3	19.6	2.05 (1.80, 2.31)*	1.90 (1.67, 2.15)*	1.99 (1.76, 2.24)*

CrI, credible interval.
*Indicates a statistically significant difference from Japan's national mean

eTable 9. Potential determinants of the number of new long-stay psychiatric patients per 100,000 population by survey year

	2016 (Overall R^2=0.92)			2015 (Overall R^2=0.91)			2014 (Overall R^2=0.89)		
Independent variable	r	B (95% CI)	$R^{2\,a}$	r	B (95% CI)	$R^{2\,a}$	r	B (95% CI)	$R^{2\,a}$
Intercept	—	−14.95 (−24.99, −4.91)*	—	—	−12.81 (−23.50, −2.11)*	—	—	−15.70 (−27.45, −3.96)*	—
1. Number of recipients of public assistance per 1,000 population	0.78	0.29 (0.12, 0.45)*	0.24	0.79	0.33 (0.15, 0.50)*	0.25	0.77	0.30 (0.10, 0.49)*	0.23
2. Number of treated patients with mental disorders per 1,000 population	0.24	0.00 (−0.08, 0.09)	0.01	0.24	0.01 (−0.08, 0.10)	0.02	0.21	−0.00 (−0.10, 0.09)	0.01
3. Number of patients receiving home visits for psychiatric nursing care per 100,000 population	0.51	−0.00 (−0.01, 0.01)	0.07	0.49	−0.00 (−0.01, 0.01)	0.07	0.51	0.00 (−0.01, 0.01)	0.08
4. Number of psychiatric beds per 100, 000 population	0.72	0.03 (0.02, 0.04)*	0.27	0.70	0.03 (0.02, 0.04)*	0.26	0.70	0.03 (0.02, 0.04)*	0.25
5. Average length of stay in psychiatric beds, days	0.27	0.01 (−0.01, 0.02)	0.04	0.25	0.01 (−0.01, 0.02)	0.03	0.28	0.01 (−0.01, 0.03)	0.04
6. Number of beds in institutions covered by long-term care insurance per 1,000 population aged ≥65 years	−0.24	0.11 (−0.20, 0.42)	0.02	−0.26	0.07 (−0.26, 0.39)	0.02	−0.25	0.14 (−0.22, 0.50)	0.02
7. Percentage of depopulated municipalities	0.39	−0.03 (−0.06, 0.01)	0.05	0.36	−0.03 (−0.07, 0.00)	0.04	0.36	−0.03 (−0.07, 0.01)	0.04
8. Percentage of population living alone	0.54	0.40 (0.10, 0.70)*	0.11	0.55	0.38 (0.06, 0.69)*	0.11	0.56	0.48 (0.13, 0.83)*	0.12
9. Unemployment rate	0.50	1.09 (−0.53, 2.71)	0.10	0.51	0.74 (−0.98, 2.47)	0.10	0.48	0.88 (−1.01, 2.77)	0.10

CI, confidence interval.
* *P*<0.05
a R^2 indicates a decomposition of overall R^2 in the multiple regression model. The sum of R^2 for all independent variables is equal to the overall R^2 in the model.

eTable 10. Age- and sex-standardized number of old long-stay psychiatric patients by prefecture and survey year, using direct and indirect methods

	Direct method, no. of admissions per 100,000 population			Indirect method, standardized claim ratio (95% CrI)		
Prefecture	2016	2015	2014	2016	2015	2014
Japan	15.8	16.5	17.2	reference	reference	reference
Hokkaido	24.8	26.2	27.8	1.55 (1.47, 1.63)*	1.56 (1.49, 1.65)*	1.59 (1.51, 1.67)*
Aomori	13.3	14.3	14.5	0.83 (0.71, 0.95)*	0.85 (0.74, 0.97)*	0.83 (0.72, 0.94)*
Iwate	11.2	11.9	12.1	0.69 (0.59, 0.81)*	0.70 (0.60, 0.81)*	0.68 (0.58, 0.79)*
Miyagi	8.9	8.9	9.6	0.56 (0.49, 0.64)*	0.53 (0.46, 0.61)*	0.55 (0.48, 0.63)*
Akita	18.6	19.0	20.5	1.15 (1.00, 1.30)*	1.13 (0.99, 1.28)	1.16 (1.02, 1.31)*
Yamagata	4.5	4.8	5.3	0.29 (0.22, 0.37)*	0.29 (0.22, 0.37)*	0.31 (0.24, 0.39)*
Fukushima	12.0	11.8	13.0	0.74 (0.65, 0.84)*	0.70 (0.61, 0.79)*	0.73 (0.64, 0.82)*
Ibaraki	16.3	16.5	17.8	1.03 (0.94, 1.12)	1.00 (0.91, 1.09)	1.03 (0.94, 1.12)
Tochigi	20.5	20.5	20.8	1.28 (1.16, 1.41)*	1.23 (1.12, 1.35)*	1.20 (1.09, 1.32)*
Gunma	7.3	7.6	8.4	0.46 (0.39, 0.54)*	0.46 (0.39, 0.53)*	0.49 (0.42, 0.56)*
Saitama	10.6	10.9	11.1	0.67 (0.62, 0.72)*	0.66 (0.61, 0.71)*	0.65 (0.60, 0.69)*
Chiba	13.3	15.2	15.3	0.83 (0.77, 0.89)*	0.91 (0.85, 0.97)*	0.88 (0.82, 0.94)*
Tokyo	19.5	20.5	20.9	1.20 (1.15, 1.25)*	1.21 (1.16, 1.26)*	1.18 (1.14, 1.23)*
Kanagawa	9.6	10.0	10.5	0.60 (0.56, 0.64)*	0.60 (0.56, 0.64)*	0.60 (0.56, 0.64)*
Niigata	10.0	10.4	10.8	0.62 (0.55, 0.70)*	0.62 (0.54, 0.70)*	0.62 (0.55, 0.70)*
Toyama	8.7	8.1	9.0	0.54 (0.44, 0.65)*	0.48 (0.39, 0.59)*	0.51 (0.42, 0.62)*
Ishikawa	11.5	11.8	13.5	0.72 (0.60, 0.84)*	0.71 (0.60, 0.83)*	0.78 (0.67, 0.91)*
Fukui	5.8	7.4	7.9	0.38 (0.28, 0.49)*	0.45 (0.35, 0.57)*	0.47 (0.36, 0.59)*
Yamanashi	8.3	9.6	9.9	0.53 (0.42, 0.66)*	0.59 (0.47, 0.72)*	0.58 (0.47, 0.71)*
Nagano	3.7	3.3	3.5	0.24 (0.19, 0.29)*	0.21 (0.16, 0.25)*	0.21 (0.16, 0.26)*
Gifu	5.6	5.8	6.0	0.35 (0.29, 0.42)*	0.35 (0.29, 0.42)*	0.35 (0.29, 0.42)*
Shizuoka	5.9	6.5	7.0	0.37 (0.33, 0.42)*	0.39 (0.34, 0.44)*	0.40 (0.36, 0.45)*
Aichi	11.3	11.5	11.9	0.71 (0.66, 0.76)*	0.70 (0.65, 0.75)*	0.70 (0.65, 0.74)*
Mie	12.3	14.2	14.7	0.77 (0.67, 0.87)*	0.85 (0.75, 0.96)*	0.85 (0.75, 0.95)*
Shiga	6.5	7.2	7.1	0.41 (0.33, 0.50)*	0.43 (0.35, 0.52)*	0.41 (0.33, 0.49)*
Kyoto	13.6	15.3	15.9	0.86 (0.77, 0.95)*	0.92 (0.84, 1.02)	0.92 (0.84, 1.01)
Osaka	19.8	20.4	21.0	1.23 (1.18, 1.29)*	1.21 (1.16, 1.27)*	1.21 (1.15, 1.26)*
Hyogo	13.3	14.1	14.4	0.83 (0.78, 0.90)*	0.84 (0.78, 0.90)*	0.83 (0.77, 0.89)*
Nara	12.6	13.0	13.6	0.79 (0.68, 0.91)*	0.78 (0.68, 0.90)*	0.79 (0.68, 0.90)*
Wakayama	13.2	13.1	13.9	0.83 (0.70, 0.97)*	0.79 (0.66, 0.92)*	0.80 (0.68, 0.94)*
Tottori	8.4	7.0	8.3	0.52 (0.39, 0.67)*	0.43 (0.32, 0.57)*	0.49 (0.37, 0.63)*
Shimane	7.3	7.4	8.8	0.47 (0.36, 0.60)*	0.45 (0.34, 0.57)*	0.51 (0.39, 0.63)*
Okayama	10.2	11.2	12.2	0.64 (0.56, 0.74)*	0.68 (0.60, 0.78)*	0.71 (0.63, 0.81)*
Hiroshima	14.6	14.4	15.3	0.91 (0.82, 1.00)*	0.86 (0.78, 0.94)*	0.88 (0.80, 0.97)*
Yamaguchi	16.0	15.6	15.3	0.99 (0.87, 1.12)	0.94 (0.82, 1.06)	0.88 (0.77, 1.00)*
Tokushima	39.9	41.8	46.2	2.46 (2.20, 2.74)*	2.49 (2.23, 2.76)*	2.61 (2.36, 2.88)*
Kagawa	19.1	19.4	20.5	1.19 (1.03, 1.36)*	1.14 (0.98, 1.30)	1.16 (1.01, 1.32)*
Ehime	13.7	13.8	14.5	0.85 (0.73, 0.97)*	0.82 (0.71, 0.94)*	0.83 (0.72, 0.95)*
Kochi	26.4	28.5	29.1	1.62 (1.41, 1.85)*	1.69 (1.48, 1.91)*	1.66 (1.45, 1.87)*
Fukuoka	37.1	38.4	39.9	2.31 (2.20, 2.42)*	2.29 (2.19, 2.40)*	2.28 (2.19, 2.39)*
Saga	20.9	21.7	22.7	1.28 (1.10, 1.48)*	1.29 (1.11, 1.48)*	1.30 (1.12, 1.48)*
Nagasaki	44.5	44.7	46.7	2.77 (2.56, 2.98)*	2.66 (2.46, 2.87)*	2.68 (2.48, 2.88)*
Kumamoto	20.0	20.9	22.0	1.25 (1.13, 1.38)*	1.26 (1.14, 1.39)*	1.27 (1.15, 1.40)*
Oita	39.5	42.0	43.1	2.46 (2.25, 2.69)*	2.50 (2.29, 2.72)*	2.46 (2.25, 2.67)*
Miyazaki	24.2	27.7	29.5	1.52 (1.35, 1.70)*	1.65 (1.48, 1.84)*	1.69 (1.52, 1.87)*
Kagoshima	39.7	41.9	43.3	2.47 (2.29, 2.66)*	2.51 (2.33, 2.69)*	2.49 (2.32, 2.67)*
Okinawa	25.9	24.3	25.1	1.69 (1.51, 1.88)*	1.53 (1.37, 1.71)*	1.50 (1.34, 1.67)*

CrI, credible interval.

*Indicates a statistically significant difference from Japan's national mean

eTable 11. Potential determinants of the number of old long-stay psychiatric patients per 100,000 population

	2016 (Overall R^2=0.88)			2015 (Overall R^2=0.88)			2014 (Overall R^2=0.88)		
Independent variable	r	B (95% CI)	$R^{2\,a}$	r	B (95% CI)	$R^{2\,a}$	r	B (95% CI)	$R^{2\,a}$
Intercept	—	−27.87 (−52.38, −3.36)*	—	—	−24.78 (−49.92, 0.36)	—	—	−27.72 (−54.34, −1.10)*	—
1. Number of recipients of public assistance per 1,000 population	0.62	0.78 (0.38, 1.19)*	0.17	0.62	0.85 (0.44, 1.27)*	0.17	0.61	0.90 (0.46, 1.34)*	0.17
2. Number of treated patients with mental disorders per 1,000 population	0.19	0.01 (−0.19, 0.22)	0.01	0.18	−0.03 (−0.24, 0.18)	0.01	0.20	0.01 (−0.21, 0.24)	0.01
3. Number of patients receiving home visits for psychiatric nursing care per 100,000 population	0.44	−0.03 (−0.05, −0.01)*	0.06	0.44	−0.04 (−0.06, −0.01)*	0.06	0.44	−0.04 (−0.06, −0.01)*	0.06
4. Number of psychiatric beds per 100, 000 population	0.73	0.06 (0.04, 0.08)*	0.30	0.73	0.06 (0.05, 0.08)*	0.30	0.74	0.07 (0.05, 0.09)*	0.31
5. Average length of stay in psychiatric beds, days	0.52	0.08 (0.04, 0.11)*	0.17	0.52	0.08 (0.04, 0.11)*	0.16	0.52	0.08 (0.04, 0.12)*	0.16
6. Number of beds in institutions covered by long-term care insurance per 1,000 population aged ≥65 years	−0.25	−0.08 (−0.83, 0.68)	0.02	−0.25	−0.14 (−0.91, 0.63)	0.02	−0.23	−0.03 (−0.85, 0.79)	0.02
7. Percentage of depopulated municipalities	0.31	−0.08 (−0.16, 0.00)	0.04	0.32	−0.08 (−0.16, 0.01)	0.04	0.33	−0.09 (−0.18, −0.00)*	0.04
8. Percentage of population living alone	0.41	0.41 (−0.32, 1.14)	0.06	0.42	0.50 (−0.25, 1.24)	0.07	0.41	0.46 (−0.33, 1.25)	0.07
9. Unemployment rate	0.32	−1.84 (−5.79, 2.11)	0.05	0.30	−2.91 (−6.96, 1.15)	0.05	0.29	−3.20 (−7.49, 1.09)	0.04

CI, confidence interval.

* *P*<0.05

a R^2 indicates a decomposition of overall R^2 in the multiple regression model. The sum of R^2 for all independent variables is equal to the overall R^2 in the model.

eTable 12. Age- and sex-standardized number of dementia-related psychiatric admissions by prefecture and survey year, using direct and indirect methods

	Direct method, number of admissions per 100,000 population			Indirect method, standardized claim ratio (95% CrI)		
Prefecture	2016	2015	2014	2016	2015	2014
Japan	3.5	3.5	3.6	reference	reference	reference
Hokkaido	6.5	6.4	6.2	1.83 (1.66, 2.02)*	1.78 (1.60, 1.96)*	1.71 (1.54, 1.88)*
Aomori	5.9	5.2	5.1	1.66 (1.33, 2.01)*	1.41 (1.12, 1.74)*	1.39 (1.10, 1.71)*
Iwate	1.5	1.5	1.8	0.45 (0.29, 0.64)*	0.41 (0.26, 0.59)*	0.49 (0.33, 0.69)*
Miyagi	3.0	3.4	3.6	0.84 (0.66, 1.05)	0.96 (0.76, 1.18)	0.99 (0.79, 1.21)
Akita	3.5	2.9	2.7	0.97 (0.71, 1.26)	0.79 (0.57, 1.05)	0.72 (0.51, 0.97)*
Yamagata	1.5	1.0	1.1	0.44 (0.27, 0.64)*	0.32 (0.18, 0.49)*	0.33 (0.19, 0.50)*
Fukushima	2.2	2.1	2.4	0.63 (0.47, 0.82)*	0.58 (0.43, 0.76)*	0.68 (0.51, 0.87)*
Ibaraki	0.9	1.0	1.2	0.26 (0.17, 0.37)*	0.29 (0.19, 0.40)*	0.35 (0.25, 0.48)*
Tochigi	1.3	1.3	1.1	0.39 (0.26, 0.55)*	0.38 (0.25, 0.54)*	0.34 (0.22, 0.48)*
Gunma	1.2	1.1	1.3	0.37 (0.24, 0.52)*	0.34 (0.22, 0.48)*	0.38 (0.26, 0.53)*
Saitama	2.0	2.2	2.0	0.55 (0.46, 0.65)*	0.62 (0.53, 0.73)*	0.54 (0.45, 0.64)*
Chiba	1.9	2.0	2.3	0.54 (0.44, 0.64)*	0.56 (0.47, 0.67)*	0.63 (0.52, 0.74)*
Tokyo	5.0	5.6	5.1	1.41 (1.30, 1.53)*	1.57 (1.45, 1.70)*	1.41 (1.30, 1.53)*
Kanagawa	3.0	2.5	2.2	0.87 (0.77, 0.99)*	0.69 (0.60, 0.79)*	0.62 (0.53, 0.71)*
Niigata	2.1	1.7	1.8	0.58 (0.44, 0.74)*	0.48 (0.35, 0.63)*	0.48 (0.36, 0.63)*
Toyama	0.7	0.5	0.8	0.23 (0.11, 0.38)*	0.18 (0.08, 0.32)*	0.26 (0.14, 0.43)*
Ishikawa	2.0	2.4	3.5	0.57 (0.37, 0.82)*	0.68 (0.46, 0.94)*	0.95 (0.69, 1.26)
Fukui	1.2	1.2	1.8	0.37 (0.19, 0.61)*	0.37 (0.19, 0.60)*	0.51 (0.30, 0.78)*
Yamanashi	0.8	1.8	1.1	0.30 (0.14, 0.51)*	0.52 (0.31, 0.79)*	0.35 (0.18, 0.57)*
Nagano	0.3	0.0	0.4	0.09 (0.04, 0.17)*	0.04 (0.01, 0.09)*	0.12 (0.06, 0.21)*
Gifu	1.0	0.7	1.2	0.31 (0.20, 0.45)*	0.23 (0.14, 0.35)*	0.35 (0.24, 0.50)*
Shizuoka	0.7	0.8	0.8	0.20 (0.13, 0.28)*	0.22 (0.15, 0.31)*	0.23 (0.16, 0.32)*
Aichi	1.8	1.8	1.9	0.52 (0.43, 0.62)*	0.51 (0.42, 0.61)*	0.53 (0.44, 0.62)*
Mie	1.8	2.3	2.2	0.52 (0.37, 0.71)*	0.64 (0.47, 0.85)*	0.61 (0.44, 0.80)*
Shiga	2.7	2.8	2.1	0.77 (0.54, 1.04)	0.78 (0.55, 1.05)	0.60 (0.41, 0.84)*
Kyoto	4.7	5.6	6.8	1.34 (1.12, 1.59)*	1.53 (1.29, 1.79)*	1.86 (1.60, 2.14)*
Osaka	5.1	5.2	5.4	1.44 (1.30, 1.58)*	1.46 (1.32, 1.60)*	1.49 (1.36, 1.63)*
Hyogo	4.0	4.0	3.6	1.13 (0.99, 1.29)	1.13 (0.98, 1.28)	0.98 (0.85, 1.12)
Nara	3.7	3.6	4.0	1.05 (0.79, 1.34)	0.99 (0.74, 1.28)	1.09 (0.83, 1.38)
Wakayama	0.9	1.3	0.7	0.29 (0.15, 0.48)*	0.38 (0.22, 0.59)*	0.24 (0.12, 0.40)*
Tottori	2.6	3.0	3.3	0.71 (0.42, 1.07)	0.85 (0.53, 1.23)	0.91 (0.59, 1.31)
Shimane	2.2	2.3	3.0	0.64 (0.39, 0.93)*	0.66 (0.41, 0.95)*	0.79 (0.52, 1.12)
Okayama	3.6	3.7	3.6	1.01 (0.79, 1.25)	1.02 (0.80, 1.26)	0.98 (0.77, 1.21)
Hiroshima	2.5	2.6	3.1	0.70 (0.55, 0.87)*	0.72 (0.56, 0.89)*	0.84 (0.68, 1.03)
Yamaguchi	3.5	3.8	3.7	0.97 (0.74, 1.23)	1.03 (0.80, 1.30)	1.02 (0.79, 1.28)
Tokushima	5.5	6.1	5.1	1.46 (1.08, 1.89)*	1.57 (1.18, 2.02)*	1.33 (0.97, 1.73)
Kagawa	2.3	2.2	2.5	0.64 (0.42, 0.91)*	0.64 (0.42, 0.90)*	0.70 (0.47, 0.97)*
Ehime	3.2	3.3	3.6	0.89 (0.67, 1.15)	0.89 (0.67, 1.15)	0.96 (0.73, 1.23)
Kochi	6.3	7.5	5.7	1.74 (1.33, 2.21)*	2.02 (1.58, 2.51)*	1.57 (1.19, 2.01)*
Fukuoka	8.8	9.1	9.8	2.48 (2.25, 2.72)*	2.51 (2.28, 2.75)*	2.66 (2.43, 2.91)*
Saga	3.2	3.3	3.4	0.92 (0.62, 1.27)	0.93 (0.64, 1.28)	0.97 (0.68, 1.32)
Nagasaki	4.7	4.5	5.2	1.32 (1.04, 1.63)*	1.24 (0.97, 1.54)	1.39 (1.11, 1.70)*
Kumamoto	5.2	4.6	4.2	1.46 (1.20, 1.75)*	1.33 (1.08, 1.60)*	1.18 (0.95, 1.43)
Oita	6.8	6.5	6.0	1.92 (1.56, 2.33)*	1.81 (1.46, 2.19)*	1.65 (1.32, 2.01)*
Miyazaki	6.3	6.4	6.2	1.74 (1.38, 2.14)*	1.79 (1.43, 2.19)*	1.72 (1.37, 2.11)*
Kagoshima	7.0	6.9	7.6	1.98 (1.66, 2.32)*	1.94 (1.63, 2.27)*	2.09 (1.77, 2.43)*
Okinawa	9.8	7.6	9.9	2.73 (2.25, 3.25)*	2.08 (1.67, 2.53)*	2.65 (2.19, 3.16)*

CrI, credible interval.

*Indicates a statistically significant difference from Japan's national mean

eTable 13. Potential determinants of the number of dementia-related psychiatric admissions per 100,000 population

	2016 (Overall R^2=0.83)			2015 (Overall R^2=0.84)			2014 (Overall R^2=0.79)		
Independent variable	r	B (95% CI)	$R^{2\ a}$	r	B (95% CI)	$R^{2\ a}$	r	B (95% CI)	$R^{2\ a}$
Intercept	—	−6.90 (−13.25, −0.54)*	—	—	−5.36 (−11.38, 0.65)	—	—	−8.08 (−15.29, −0.87)*	—
1. Number of recipients of public assistance per 1,000 population	0.81	0.08 (−0.02, 0.19)	0.25	0.83	0.11 (0.02, 0.21)*	0.26	0.78	0.08 (−0.04, 0.20)	0.23
2. Number of treated patients with mental disorders per 1,000 population	0.14	−0.02 (−0.08, 0.03)	0.01	0.18	−0.02 (−0.07, 0.03)	0.01	0.12	−0.03 (−0.09, 0.03)	0.01
3. Number of patients receiving home visits for psychiatric nursing care per 100,000 population	0.44	0.00 (−0.00, 0.01)	0.07	0.43	0.00 (−0.00, 0.01)	0.05	0.45	0.00 (−0.00, 0.01)	0.07
4. Number of psychiatric beds per 100, 000 population	0.56	0.01 (0.00, 0.01)*	0.15	0.55	0.01 (0.00, 0.01)*	0.15	0.53	0.01 (0.00, 0.01)*	0.13
5. Average length of stay in psychiatric beds, days	0.10	−0.00 (−0.01, 0.01)	0.01	0.10	−0.00 (−0.01, 0.01)	0.01	0.10	−0.00 (−0.01, 0.01)	0.01
6. Number of beds in institutions covered by long-term care insurance per 1,000 population aged ≥65 years	−0.27	0.04 (−0.16, 0.23)	0.02	−0.30	0.01 (−0.17, 0.20)	0.03	−0.24	0.11 (−0.11, 0.34)	0.02
7. Percentage of depopulated municipalities	0.31	0.00 (−0.02, 0.02)	0.04	0.31	−0.00 (−0.02, 0.02)	0.03	0.29	−0.00 (−0.03, 0.02)	0.03
8. Percentage of population living alone	0.54	0.16 (−0.03, 0.35)	0.10	0.63	0.23 (0.05, 0.41)*	0.15	0.57	0.25 (0.04, 0.47)*	0.13
9. Unemployment rate	0.65	1.59 (0.57, 2.62)*	0.20	0.59	0.73 (−0.24, 1.70)	0.14	0.60	1.31 (0.15, 2.47)*	0.16

CI, confidence interval.
* *P*<0.05
[a] R^2 indicates a decomposition of overall R^2 in the multiple regression model. The sum of R^2 for all independent variables is equal to the overall R^2 in the model.

eTable 14. Differences in psychiatric admission data between the FSMA and the 630 survey

Prefecture	Direct method, number of admissions per 100,000 population in the 630 survey				Differences in psychiatric admissions covered by public assistance per 100,000 population between the actual and expected number of admissions			
	Total	New long-stay	Old long-stay	Dementia	Total	New long-stay	Old long-stay	Dementia
Japan	224.1	65.4	76.8	52.0	—	—	—	—
Hokkaido	296.3	86.7	96.0	76.6	24.9	7.0	8.3	2.3
Aomori	272.9	76.9	78.7	74.4	4.3	2.1	0.3	1.8
Iwate	271.1	73.7	99.5	40.4	–8.6	–0.8	–6.0	–0.7
Miyagi	230.2	75.1	70.7	71.5	–7.5	–1.1	–2.4	–1.0
Akita	309.0	87.7	103.2	81.7	–1.8	–0.5	0.6	–1.0
Yamagata	266.2	78.5	72.9	69.0	–18.5	–4.0	–7.3	–2.3
Fukushima	254.3	79.3	93.7	44.2	–10.7	–2.8	–4.0	–0.3
Ibaraki	208.0	54.5	90.3	31.0	1.9	0.7	1.0	–0.9
Tochigi	224.8	63.2	98.9	34.0	5.7	0.4	3.4	–0.6
Gunma	232.7	63.7	95.2	35.1	–15.6	–3.8	–9.0	–0.8
Saitama	185.6	56.1	57.3	65.1	0.5	–0.7	2.0	–1.6
Chiba	154.5	44.4	63.4	28.9	7.4	1.2	3.5	0.2
Tokyo	160.9	45.1	44.9	33.4	27.9	7.9	13.5	3.1
Kanagawa	116.4	33.1	31.9	27.7	15.9	4.7	6.3	1.4
Niigata	239.6	66.0	93.1	50.8	–9.8	–1.6	–5.9	–0.8
Toyama	261.5	78.6	96.3	51.8	–20.0	–5.7	–7.9	–2.2
Ishikawa	289.9	87.5	95.5	74.4	–15.6	–3.8	–4.9	–2.1
Fukui	247.8	66.5	81.7	69.9	–20.7	–4.2	–7.8	–2.7
Yamanashi	225.7	57.7	88.1	30.9	–12.3	–3.8	–6.6	–0.9
Nagano	186.1	52.5	58.6	27.0	–11.9	–2.7	–5.1	–1.3
Gifu	165.6	39.7	64.4	22.6	–8.6	–2.0	–4.4	–0.3
Shizuoka	152.4	39.1	56.7	23.2	–3.4	–0.5	–2.5	–0.7
Aichi	155.6	41.1	58.8	21.4	5.8	1.4	2.4	0.5
Mie	226.1	65.7	86.5	45.0	–3.7	–2.0	–2.3	–0.7
Shiga	152.0	45.1	50.2	42.5	–4.0	–2.3	–0.6	0.3
Kyoto	155.2	49.7	49.0	60.4	15.3	2.0	6.7	1.4
Osaka	193.6	55.4	60.2	45.6	21.8	4.9	10.6	2.5
Hyogo	188.2	53.6	64.0	41.2	9.4	2.2	3.4	1.7
Nara	176.4	48.8	54.4	43.4	6.7	1.2	4.6	1.3
Wakayama	168.9	45.0	73.1	10.3	6.0	1.1	1.4	0.2
Tottori	270.4	85.3	82.2	73.7	–12.9	–3.4	–5.3	–1.4
Shimane	271.4	78.6	82.1	48.9	–18.4	–3.0	–6.4	–0.5
Okayama	230.9	71.4	65.1	66.8	–2.4	–0.8	0.0	–0.1
Hiroshima	276.6	84.3	92.9	63.9	–5.9	–1.9	–1.3	–1.1
Yamaguchi	356.8	117.4	120.5	94.1	–11.8	–2.5	–5.5	–1.6
Tokushima	405.2	107.3	197.5	44.6	13.5	3.8	2.7	3.0
Kagawa	293.2	89.3	107.7	56.0	–0.3	–1.8	0.2	–0.8
Ehime	274.8	77.2	101.3	54.3	–1.1	1.1	–3.9	0.2
Kochi	368.8	114.5	112.6	88.6	19.3	7.1	6.5	1.5
Fukuoka	378.9	119.9	128.5	103.7	22.0	5.8	14.0	3.1
Saga	451.2	150.0	137.9	117.6	–19.8	–5.6	–4.2	–3.1
Nagasaki	473.8	140.5	184.3	93.4	14.4	2.7	9.9	–0.4
Kumamoto	431.4	123.9	154.3	107.1	–11.8	–3.0	–8.4	–0.7
Oita	392.9	114.9	153.7	92.9	16.5	2.4	11.2	1.7
Miyazaki	454.1	134.9	169.1	117.5	–9.1	–1.2	–7.3	–0.1
Kagoshima	508.9	148.1	199.3	119.8	5.8	0.9	2.1	0.5
Okinawa	402.1	123.2	120.5	99.5	20.8	4.8	4.4	4.4
